# Supplementary material for: Blocking transport resonances via Kondo many-body entanglement in quantum dots
Source: Nat Commun. 2016 Aug 16;7:12442. doi: 10.1038/ncomms12442 (PMC4990698; doi:10.1038/ncomms12442)
Supplement: Supplementary Information — Supplementary Figures 1-8, Supplementary Table 1, Supplementary Notes 1-5, Supplementary Methods and Supplementary References [file ncomms12442-s1.pdf]

Supplementary Figures

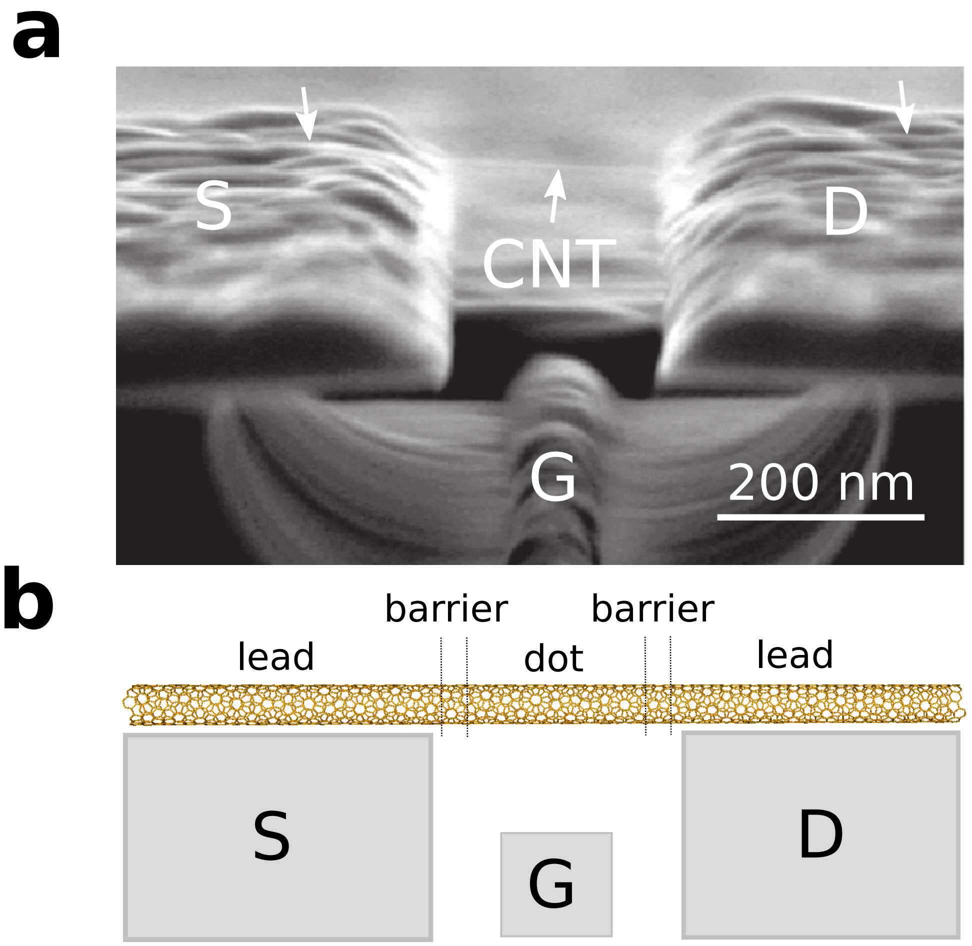

Supplementary Figure 1: Experimental device and schematics. **a,** Scanning electron microscopy of an ultraclean carbon nanotube based device similar to the one used in the experiment. A carbon nanotube (CNT) is suspended on top of litographically fabricated contacts. **b,** Schematic of the suspended CNT device. A segment of the CNT acts as a quantum dot separated by tunneling barriers from the rest of the tube. These latter parts of the nanotube act as electronic reservoirs from which charge carriers can tunnel into and out of the dot. The separation between the contacts defines the size of the quantum dot while the local back gate voltage influences the electrostatic profile seen by the charge carriers.

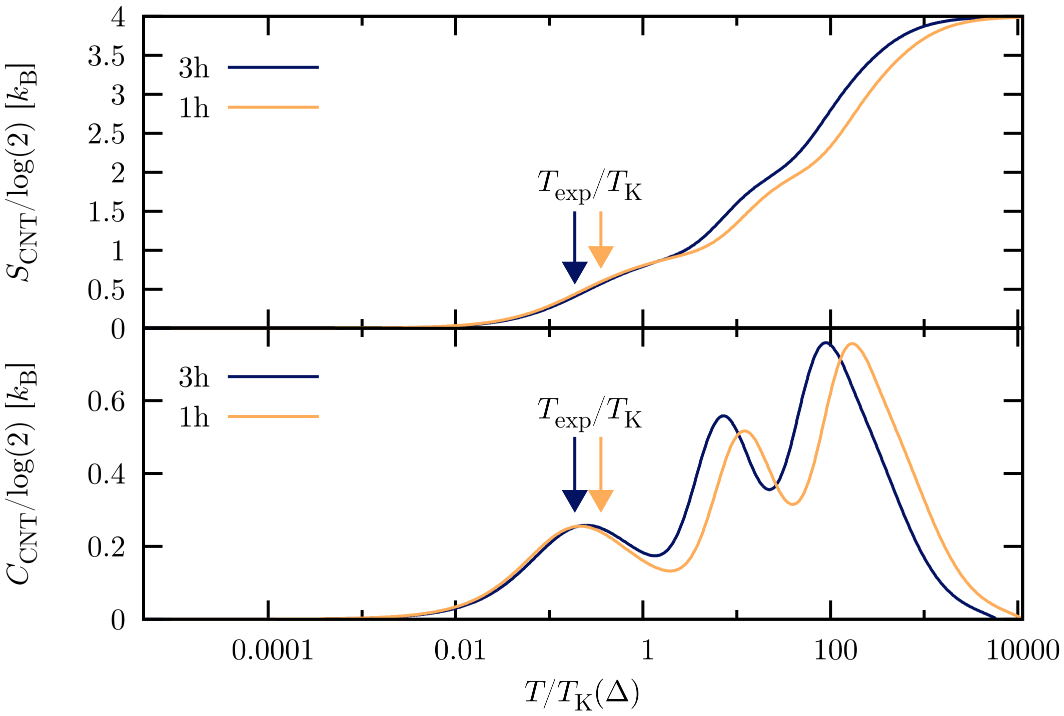

Supplementary Figure 2: Entropy and specific heat. Evolution of the impurity entropy and specific heat as a function of the temperature. The entropy is zero at zero temperature, indicating a non degenerate ground state. At finite temperatures, it remains close to zero up to temperatures of the order of 10^{-2} $T_{\rm K}$, indicating that the system is to a good approximation in the ground state. At higher temperatures thermal fluctuations become more important but universality is preserved up to temperatures of the order of $T_{\rm K}$, where a shoulder is observed. For even larger temperatures the upper Kramers charge state becomes accessible, yielding a shoulder in the vicinity of Δ; finally, a plateau with value $4k_{\rm B}\log 2$ is observed at about $U/2$. In the correspondence of such shoulders and of the plateau the specific heat has local minima.

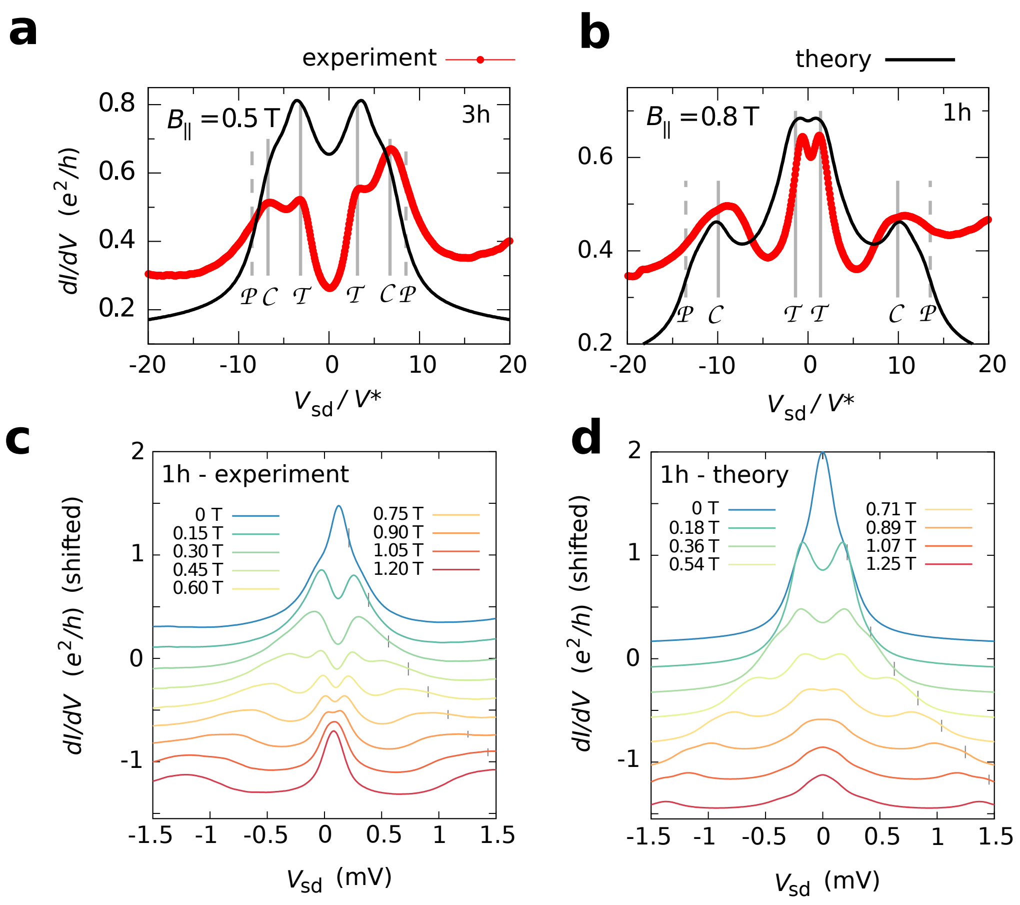

Supplementary Figure 3: Differential conductance at several magnetic field amplitudes. Bias traces of the differential conductance at various magnitudes of an applied axial magnetic field in the valence shell $N_h = 6$. **a** and **b,** Traces for the 3h and 1h valleys, respectively. The bias voltage is scaled by the voltage V^* at which the differential conductance at zero applied field reaches 0.8 of the conductance at zero bias. The vertical lines denote the position of the $\mathcal{P}$ and $\mathcal{C}$ resonances as expected from the addition spectrum. **c** and **d,** Experimental and theoretical bias traces for different values of the magnetic field. The qualitative evolution of the bias traces is similar in the theory and experiment, despite the shapes differ. The short vertical lines denoted the expected position of the $\mathcal{P}$ resonance, which is not visible at low fields, neither in the theoretical nor in the experimental traces. However, a signature of such resonance is observed at fields of about 0.9 T, indicating that Kondo screening has been weakened by the bias voltage necessary to induce a $\mathcal{P}$ transition.

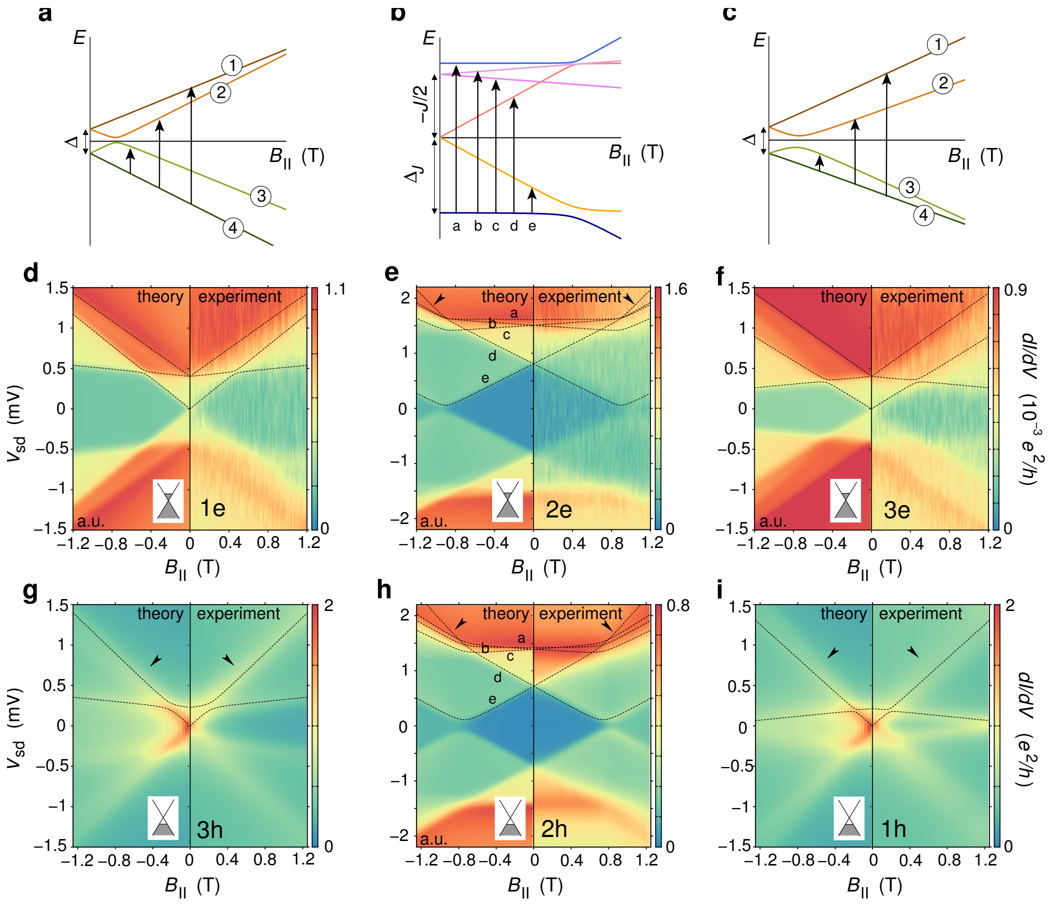

Supplementary Figure 4: Energy spectrum and differential conductance in parallel magnetic field. **a-c**, Excitation spectra for a longitudinal mode with electron filling (from left to right) $n_e = 1$ (1 electron, 3 holes), $n_e = 2$ (2 electrons, 2 holes) and $n_e = 3$ (3 electrons, 1 hole). **d-f**, dI/dV in the electron regime for gate voltages in the middle of the 1e, 2e and 3e charge states of the shell N_e. Each panel reports experimental data (positive magnetic field) and transport calculations (negative field). The dotted lines correspond to the transition energies from the ground state calculated directly from the spectra a)- c). The missing resonances are indicated by arrows. **g-i**, Panels analogous to the ones in d)-f), related to the N_h shell in the hole regime.

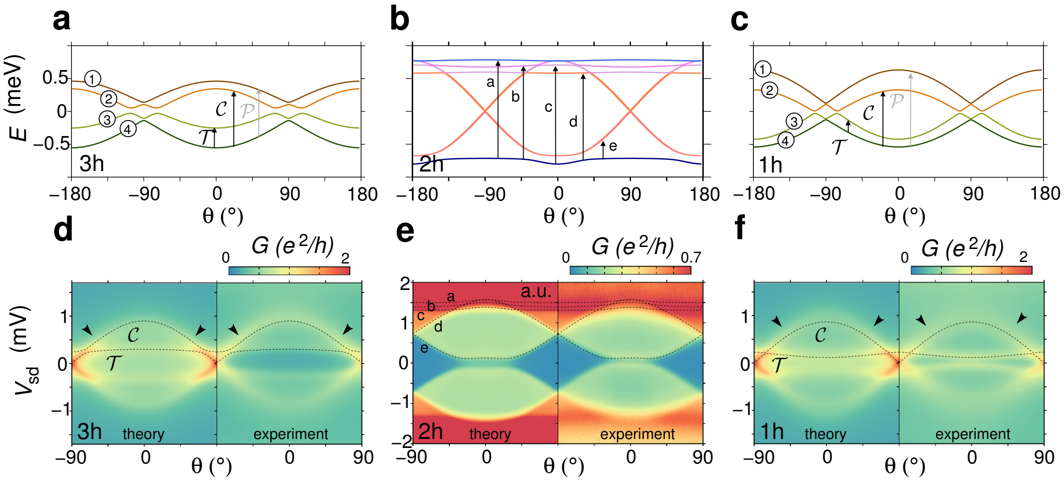

Supplementary Figure 5: Dependence on magnetic field orientation. Angular dependence of the energy spectrum and $\mathrm{d}I/\mathrm{d}V$ characteristics for negative gate voltages in the hole sector (shell N_{h}). **a-c,** Calculated energy level spectrum at fixed magnetic field, as a function of the angle θ between tube axis and the direction of the magnetic field. **d-f,** Corresponding as-measured (right hand side) and calculated differential conductance maps (left hand side) in the hole regime with respective hole fillings 3h, 2h and 1h (from left to right). The missing $\mathcal{P}$ resonances are indicated by arrows. The panels a)-f) correspond to an applied magnetic field of 0.8 Tesla.

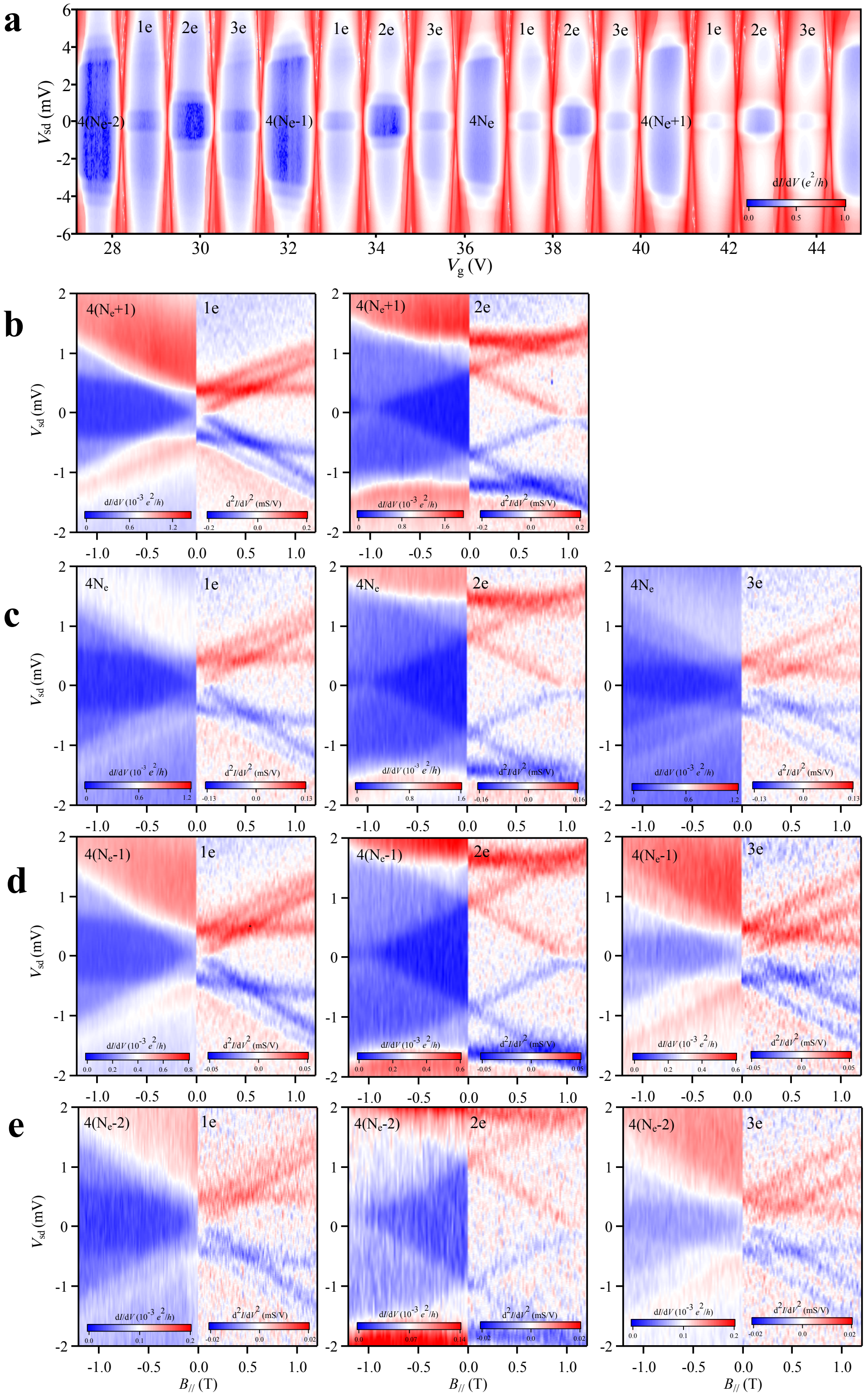

Supplementary Figure 6: Magnetotransport data in the cotunneling regime for different electron quadruplets. **a,** As-measured $\mathrm{d}I/\mathrm{d}V$ stability diagram at $B_{\parallel}=0$, representing the successive filling of up to four consecutive electron shells. The data discussed in the main text correspond to the quadruplet N_e with $4N_\mathrm{e}$ total electrons in the conduction band. **b-e,** Magnetotransport measurements of the 1e, 2e and 3e charge states (from left to right) for electrons in the quadruplets $N_\mathrm{e}+1$ (b), N_e (c), $N_\mathrm{e}-1$ (d) and $N_\mathrm{e}-2$ (e), as a function of V_{sd} and $B_{\parallel}$. In each panel (b-e), we plotted both the experimental $\mathrm{d}I/\mathrm{d}V$ ($B_{\parallel}<0$) and its numerical derivative $\mathrm{d}^2I/\mathrm{d}V^2$ ($B_{\parallel}>0$). We notice here the extreme qualitative similarity of the magnetotransport curves, highlighting the robustness of the energy level spectrum, reproducible for several electron quartets.

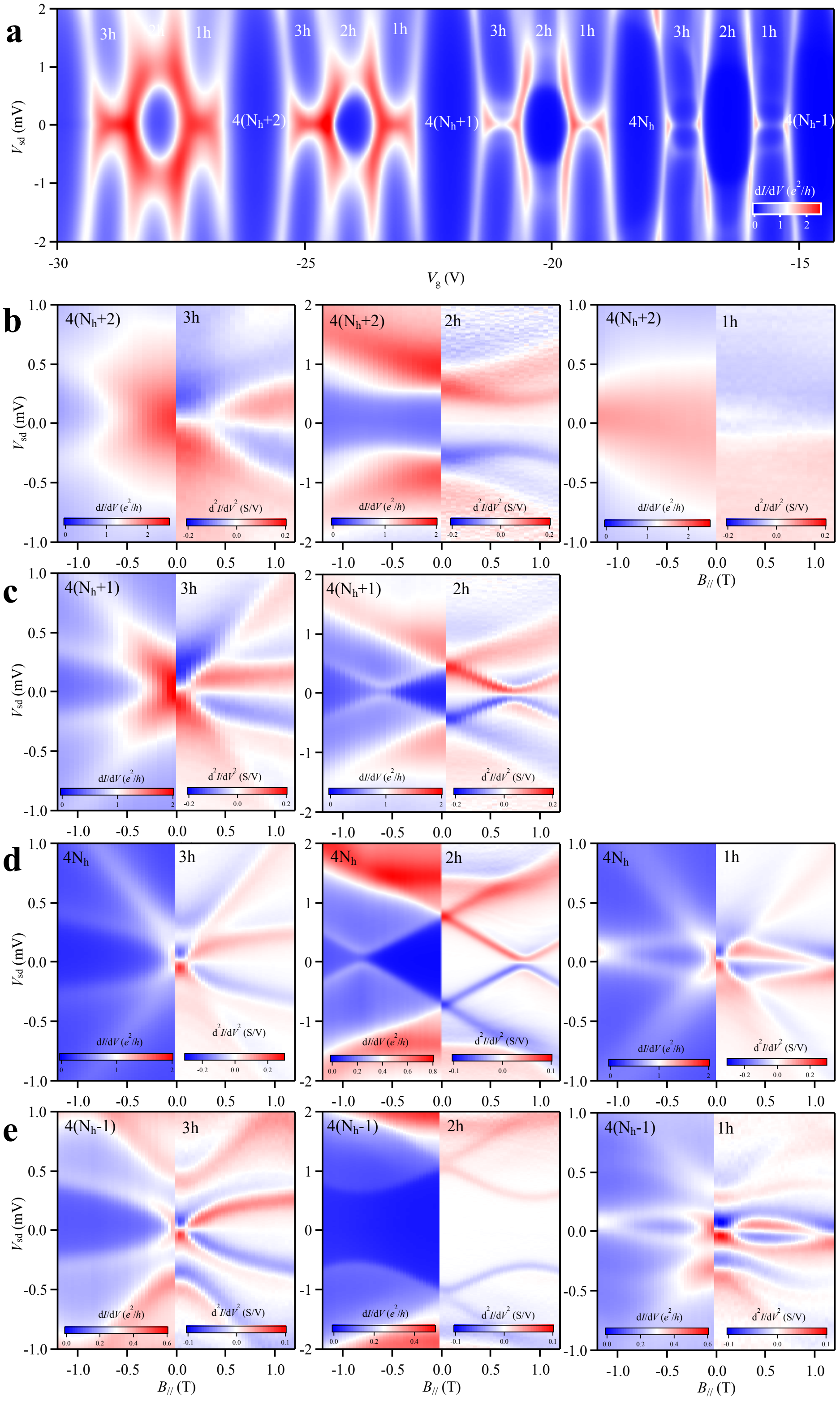

Supplementary Figure 7: Magnetotransport data for different hole quartets. **a,** As-measured dI/dV stability diagram at $B_{\parallel}$=0, representing the successive filling of up to four consecutive hole quadruplets. The data discussed in the main text correspond to the hole quartet $N_{\rm h}$. **b-e,** Magnetotransport measurements of the 3h, 2h and 1h charge states (from left to right) for hole shells $N_{\rm h}$+2 (b), $N_{\rm h}$+1 (c), $N_{\rm h}$ (d) and $N_{\rm h}$-1 (e), as a function of $V_{\rm sd}$ and $B_{\parallel}$. In each panel (b-e), we plotted both dI/dV ($B_{\parallel} < 0$) and d^2I/dV^2 ($B_{\parallel} > 0$) for direct comparison of both representations. By applying more negative gate voltage (shells $N_{\rm h}$+1 and $N_{\rm h}$+2), Kondo correlations are strengthened and the system approaches more and more the $SU(4)$ Kondo regime. For all quadruplets similar conclusions regarding the absence of $\mathcal{P}$ resonances in the Kondo regime can be drawn.

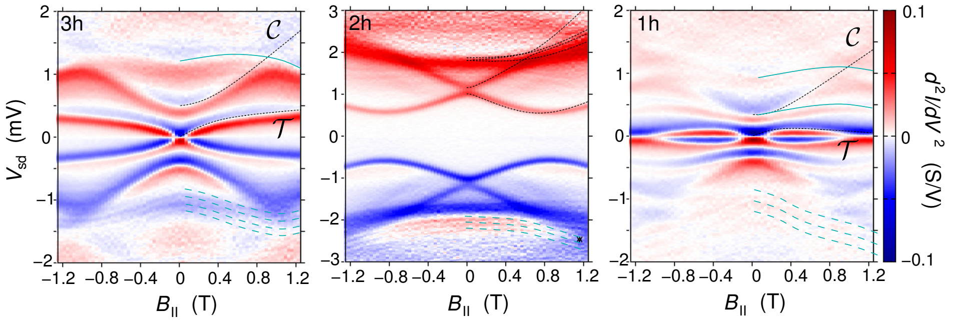

Supplementary Figure 8: Magnetotransport curves for the N_h-1 hole quartet with superposed excitation spectrum lines. Similar to the N_h quadruplet (cf. Fig. 2 of main manuscript), an analysis of the excitation spectrum allows us to identify the low energy $\mathcal{T}$ and $\mathcal{C}$ resonances at 1h and 3h filling. Additional harmonic excitations are also seen, which are not captured by our model Hamiltonian, for all hole fillings (dashed and solid lines).

Supplementary Tables

shell		Δ_{SO}(meV)	$\Delta_{KK'}$(meV)	μ_{orb}(meV T^{-1})	J(meV)
electrons	N_e+1	−0.40	0.04	0.37	−1.05
	N_e	−0.40	0.04	0.40	−1.30
	N_e-1	−0.40	0.04	0.45 (1e, 3e), 0.32 (2e)	−1.00
	N_e-2	−0.42	0.04	0.48	−1.80
holes	N_h+2	−0.21	0.30 (2h), 0.35 (3h)	0.43	−1.10
	N_h+1	−0.21	0.08	0.48	−1.10
	N_h	−0.21	0.08	0.55 (1h), 0.51 (2h, 3h)	−1.30
	N_h-1	−0.21 (1h, 2h), −0.27 (3h)	0.27 (1h), 0.38 (2h, 3h)	0.70 (1h), 0.53 (2h), 0.60 (3h)	−1.65

Supplementary Table 1. The parameters used to fit the transport spectra of the CNT at various shells and filling numbers. The experimental data show an offset in V_{sd} of 0.28 meV(0.12 meV) and are tilted in the magnetic field by −0.06 meV T^{-1} (−0.05 meV T^{-1}) at the electron(hole) side. The hole side has an offset of the magnetic field of 0.06 T.

Supplementary Methods

The total Hamiltonian

In this section we discuss our theoretical modeling of the CNT-leads complex. A scanning electron microscopy of a device similar to the one used in the experiment is shown in the Supplementary Figure 1. In order to capture Coulomb blockade and Kondo physics, a segment of the CNT forming the quantum dot is assumed to be weakly coupled by a tunneling Hamiltonian to biased source (s) and drain (d) leads. The model Hamiltonian is then given by

$$\hat{H}_{\mathrm{tot}} = \hat{H}_{\mathrm{CNT}} + \hat{H}_{\mathrm{tun}} + \hat{H}_{\mathrm{L}}, \tag{1}$$

where $\hat{H}_{\mathrm{CNT}}$ is the Hamiltonian of the isolated CNT reported in Eq. (2) of the Methods. In particular, its single particle part $\hat{H}_0 = \hat{H}_{\mathrm{d}} + \hat{H}_{\mathrm{KK'}} + \hat{H}_{\mathrm{SO}} + \hat{H}_{\mathrm{B}}$ is diagonal in the Kramers basis spanned by the quadruplet $\{|i\rangle\}$, $i = 1, 2, 3, 4$ (shown in Fig. 1b of the main manuscript). Finally, $\hat{H}_{\mathrm{tun}}$ and $\hat{H}_{\mathrm{L}}$ are the tunneling and leads Hamiltonians, respectively. In what follows a diagonal tunneling coupling is assumed for the tunneling Hamiltonian in the Kramers basis:

$$\hat{H}_{\mathrm{tun}} = \sum_{l=\mathrm{s,d}} \sum_{k,i} (T_{lki} \hat{c}^{\dagger}_{lki} \hat{d}_i + h.c.), \tag{2}$$

with k an index describing a continuum of modes in the leads. As suggested by the Supplementary Figure 1, we assume that the dot is a segment of the CNT and that the Kramers index i is conserved during the tunneling. To this Hamiltonian the coupling constants $\Gamma_{li} \equiv 2\pi\rho_l(\varepsilon_{\mathrm{F}})|T_{lki}|^2$ are associated, where $\rho_l(\varepsilon_{\mathrm{F}})$ is the density of states of lead l at the Fermi energy, and only a dependence on the lead l and Kramers index i has been retained in the tunneling coupling T_{lki}. In absence of SOC, valley mixing and exchange interaction the total Hamiltonian is $SU(4)$ invariant when all the couplings related to the lead l are equal: $\Gamma_{li} = \Gamma_l$ [1, 2], while the symmetry becomes $SU(2)\otimes SU(2)$ when only the rates within a Kramers channel are the same [3]. Finite valley scattering and SOC also reduce the symmetry to $SU(2)\otimes$ $SU(2)$ [4, 5]. We assumed equal couplings within the Kramers channels $\Gamma_{l1} = \Gamma_{l2} = \Gamma_{l\mathrm{u}}$, $\Gamma_{l3} = \Gamma_{l4} = \Gamma_{l\mathrm{d}}$ in the DM-NRG simulations, and equal rates, $\Gamma_{l\mathrm{u}} = \Gamma_{l\mathrm{d}} = \Gamma_l = \Gamma$ for the Keldysh effective action (KEA) case.

An additional exchange coupling further breaks the $SU(2)$ symmetries in the 2h valley. For our DM-NRG calculations in the Kondo regime we have neglected the exchange coupling J. Its inclusion would have been possible but at the expense of a significant increase in the computational cost, due to the reduced symmetry. Likewise J was not included in the KEA.

Supplementary Notes

Supplementary Note 1. Impurity entropy and specific heat

One outcome of the experiment is that Kondo correlations, leading to the formation of the Kondo singlets, persist also at the finite temperature of the experiment. We notice that the mixing chamber temperature $T_{\mathrm{exp}} = 30$ mK sets a lower bound to the actual experimental temperature. Given the uncertainty in determining the experimental Kondo temperature from the width of the zero bias peak of the differential conductance (cf. Supplementary Figure 2 and related discussion), we can only give a range for the ratio T/T_{K} in the experiment. According to our DM-NRG calculations for the linear conductance (cf. Fig. 1h of the main manuscript), it lies in the range $0.1 < T/T_{\mathrm{K}} < 1$. To further quantify the impact of thermal fluctuations, we have performed additional calculations of the impurity entropy and specific heat. Thermodynamic entropies $S(T) = k_{\mathrm{B}} \ln Z(T) + \langle \hat{H} \rangle / T$, associated to a given Hamiltonian $\hat{H}$, are easily accessible through DM-NRG schemes [6–9]. Here Z is the partition function and $\langle ... \rangle$ the associated thermal average. Specifically, we have calculated the conditional entropy $S_{\mathrm{CNT}}(T) = S_{\mathrm{tot}}(T) - S_{\mathrm{L}}(T)$ as a function of temperature. This so called impurity entropy is zero if the total system is in a pure singlet ground state (degeneracy one). At finite temperature also excited states become accessible and the conditional entropy $S_{\mathrm{CNT}}(T)$ grows. As seen in the Supplementary Figure 2, it grows very slowly in the Fermi liquid regime which persists up to temperatures of the order of $10^{-2}T_{\mathrm{K}}$. At higher temperatures the growth is faster but the behavior is still universal until $T \approx T_{\mathrm{K}}$. At even larger temperatures nonuniversal features are seen, like a shoulder at temperatures of the order of Δ/k_{B}. At very high temperatures the CNT is in a fully incoherent mixture, where all of its Fock states are occupied. Correspondingly, the entropy shows a plateau with value $S_{\mathrm{CNT}}/k_{\mathrm{B}} = \log(16)$. A second thermodynamic quantity of interest is the specfic heat $C = T\partial S/\partial T$. It can be calculated numerically within the Budapest code from the relation $C(T)/k_{\mathrm{B}} = \beta^2 \langle (\hat{H} - \langle \hat{H} \rangle)^2 \rangle$ [8]. The impurity specific heat $C_{\mathrm{CNT}} = C_{\mathrm{tot}} - C_{\mathrm{L}}$ is reported in the bottom panel of Fig. S2. Minima of C_{CNT} are clearly resolved in correspondence of the position of the shoulders and of the plateau of S_{CNT}. The results shown in the Supplementary Figure 2 have been obtained using a constant number of multiplets $N_{\mathrm{kept}} = 8192$ (fixing the initial discarding iteration to $m_0 = 2$) and averaged the results, according to Ref. [6], over $n_z = 4$ simulations with $z = 1/4, 1/2, 3/4, 1$ and the discretization parameter $\Lambda = 5$.

Supplementary Note 2. Transport regimes

All calculations shown in the main part of the paper have been performed using the total Hamiltonian (1) with a different set of parameters and computational techniques depending on the transport regime. In the experiment several electron and hole longitudinal modes can be investigated by sweeping the gate voltage. According to the notation used in the Supplementary Figures 4 and 5, the data shown in the main text refer to the conduction quadruplet N_e and to the valence quadruplet N_h, respectively. As shown in Table 1 of the main manuscript, the parameters Δ_SO, $\Delta_\mathrm{KK'}$ and μ_orb entering the single-particle Hamiltonian $\hat{H}_0$ do not differ much for the electron and hole sectors. However, the charging energy U greatly vary in the two regimes (but not the couplings Γ_{li}), which requires the use of different transport methods.

Weak coupling regime. At positive gate voltages (electron regime) transport is dominated by sequential and cotunneling processes, as clearly observed in the experimental transport data of Fig. 1c, 1e. This indicates large charging energies U and a parameter regime where Kondo correlations are not relevant yet. A perturbative calculation in the couplings Γ_{li} is expected to qualitatively capture the dominant sequential tunneling and cotunneling mechanisms [10]. For our transport calculations we have followed a reduced matrix density approach which retains all tunneling processes up to second order in the couplings Γ_{li} [11]. As seen from the comparison shown in Figs. 1,2 of the main manuscript, this approach indeed well captures all the inelastic features observed in the experiment in the whole electron sector, as well as for the 2h case. Due to its perturbative nature though, it fails to account for a broadening of the conductance traces being larger than the nominal experimental temperature (a thermal broadening is expected from the PT), see Fig. 1e. This large broadening signals that also higher order charge fluctutation processes might influence transport, yielding a width governed by Γ rather than by the temperature [12–15]. From the experimental curves we extract an average half width at half maximum of 0.15 meV.

In the perturbative simulations, much smaller values of the tunneling coupling had to be choosen in order to fullfill the theoretical requirement $\Gamma \ll k_\mathrm{B}T$ for the application of the PT. Since the calculations also include an exchange splitting, it was convenient in this regime to work in the $\{|\tau,\sigma\rangle\}$ basis. Besides the parameters shown in Table 1 of the main manuscript, the additional parameters used in the simulations are $T = 232$ mK, and symmetric leads with tunneling couplings $\Gamma_{K,\uparrow} = \Gamma_{K',\downarrow} = 0.06\mu$eV, $\Gamma_{K,\downarrow} = \Gamma_{K',\uparrow} = 0.047\mu$eV.

Strong coupling regime. The appearance of Kondo ridges in valleys with odd hole occupations indicates a regime of strong correlations which requires a nonperturbative treatment in the couplings Γ_{li}.

To quantitatively analyze the linear regime, we have performed numerical DM-NRG calculations using the open access Budapest code [16]. We have assumed a broken $SU(4)$ symmetry, and used the generators of the $SU(2)$ symmetries associated to the upper and lower Kramers channels along the lines explained in Sec. 2 of [5]. Such calculations, performed at $T = 30$ mK and with the parameters of the isolated CNT given in Table 1 of the Methods, enabled us in particular to estimate the coupling to the leads and the charging energy U. We allow for an asymmetry $\alpha = 4\tan\gamma/(1+\tan\gamma)^2$ between source (s) and drain (d) leads, with $\tan\gamma = \Gamma_{\mathrm{d}i}/\Gamma_{\mathrm{s}i}$ (which fixes the height of the 2-3 peak in the gate trace of Fig. 1f). The fit in Fig. 1f yields: $\alpha = 0.9$ and $\Gamma_\mathrm{su} = 0.50$ meV, $\Gamma_\mathrm{sd} = 0.64$ meV for the u/d Kramers channels coupled to the s-lead. Furthermore, we find $U = 4.7$ meV, which in turn yields the Kondo temperatures $T_\mathrm{K}(\Delta)$, as shown in Fig. 1h. Notice that a different choice of the temperature T would have implied a different set of parameters for fitting to the experiment, and hence also different Kondo temperatures. Nevertheless, we are confident that our estimate of the ratio T/T_K is close to the experimental one, as is this ratio, for example, which determines how pronounced are the minima of the linear conductance in valley 1h and 3h.

In order to describe also finite bias effects, and hence account for the inelastic transitions, one has to resort to transport approaches to the Kondo effect valid out of equilibrium. To this extent we have used the Keldysh effective action (KEA) approach, recently developed by some of us [17, 18]. The KEA is based on a slave boson field integral formulation, where a truncation of the effective action to terms quadratic in the slave boson fields enables us to evaluate the tunneling density of state of the Kondo quantum dot in analytic form. Due to such truncation, some cotunneling terms which become relevant at high bias voltage or large magnetic fields are neglected. As such, the KEA is expected to quantitatively describe the positions of the inelastic Kondo peaks at finite bias and magnetic field, but only qualitatively their shapes [18].

For the KEA calculations symmetric and equal couplings $\Gamma_{li} = \Gamma$ were assumed, an infinite charging energy U and no exchange coupling. This parameter choice necessarily yields a different Kondo temperature than in the experiments, which e.g. naturally have a finite charging energy and different couplings $\Gamma_{l\mathrm{u}}$ and $\Gamma_{l\mathrm{d}}$. Moreover, due to the choice of infinite U, our calculations well describe Kondo behavior when the effects of the fluctuations to states with double occupancy can be neglected, i.e. away from the middle of the diamonds with one or three holes. The experimental curves shown in the Figs. 2 and 4 of the main text, however, correspond to gate voltages in the middle of the 1h and 3h diamonds. Nevertheless, due to the universal scaling properties of the differential conductance in the Kondo regime, the predictions of the KEA theory can become quantitative when both the experimental data and the theoretical curves are scaled by

the respective Kondo voltage [19], or by an energy scale proportional to it (cf. next Supplementary Note 4), as long as only universal features are relevant.

Supplementary Note 3. Differential conductance traces

Experimental bias traces are compared to (zero temperature) KEA predictions in the Supplementary Figure 3. An overall qualitative agreement between theoretical and experimental prediction is observed. A quantitative agreement is found regarding the position of the $\mathcal{T}$ and $\mathcal{C}$ resonances, see e.g. panels a) and b). Clearly, the $\mathcal{P}$ resonance, whose expected position is indicated by vertical lines, is not resolved at low fields. Signatures of the re-emergence of such transition are observed at fields of the order of 0.9 T, a shoulder in panels c) and d). However, higher magnetic fields, not accessible to the experiment, would be necessary to track the evolution of this shoulder at even higher bias voltages. In our calculations the temperature was set to zero and we have choosen a source drain voltage V^* such that $G(T=0, V=V^*) = 0.8G(T=0, V=0)$. At this voltage, the dynamics is still universal and V^* is proportional to the Kondo voltage. E.g. for our symmetric set-up with $\Gamma_{li} = \Gamma$ we find $V^* = 0.41V_\text{K}$ with $V_\text{K} = k_\text{B}T_\text{K}/e$. We have also checked the evolution of this relation at finite temperatures. For temperatures $T = 0.13\Delta/k_\text{B}$, similar to the experiment, we found $G(T, V=V^{**}) = 0.8G(T, V=0)$ with $V^{**} = 0.59V_\text{K}$. To allow for comparison, the experimental data are scaled by a bias voltage $V^*_\text{exp} = 0.0842$ mV for which $G(T, V=V^*_\text{exp}) = 0.8G(T, V=0)$ for both the 1h and 3h valleys.

Supplementary Note 4. Differential conductance stability diagrams

This section provides additional experimental data, with their theoretical analysis, complementary to the ones presented in the main text. The Supplementary Figure 4 reports the as-measured and simulated magnetospectra of the differential conductance $\mathrm{d}I/\mathrm{d}V$, used to infer the current second derivative $\mathrm{d}^2I/\mathrm{d}V^2$ plots in Fig. 2 of the main text by numerical derivation, in both cotunneling and Kondo regimes. The inflexion points of the differential conductance steps (Supplementary Figure 4), in the cotunnelling regime, thus translate into extrema in the $\mathrm{d}^2I/\mathrm{dV}^2$ (Fig. 2). Analogously, maxima of the differential conductance at the Kondo resonance peaks appear as zeros of the $\mathrm{d}^2I/\mathrm{d}V^2$. In the Supplementary Figure 5 we provide the differential conductance version of the energy spectrum angular dependence (cf. Fig. 4 main text), including also the 2h valley. The same conclusions regarding the nature of the relevant inelastic resonances can be drawn, as obtained from the inspection of the current second derivative plots of the main text, used because of enhanced eye visibility of the excitation spectra.

Supplementary Note 5. Magnetospectrum of other quartets

Up to now, we only considered one selected quartet for holes and electrons lying respectively in the CNT valence and conduction bands. Here we show that comparable conclusions regarding the suppression of $\mathcal{P}$ channels due to Kondo screening can be drawn for other quartets, showing the generality of the analysis depicted in the main text.

In the cotunneling regime, observed for electrons lying in the conduction band, the CNT transport characteristics has been thoroughly investigated by magnetospectroscopy for different electron fillings. The Supplementary Figure 6 depicts part of the measurements, which display the same qualitative behavior as the studies in Fig. 2 of the main text.

Analogously, the strongly correlated regime has been investigated for various hole shell numbers (see additional data in the Supplementary Figure 7). By tuning the gate voltage it is possible to follow the system evolution over the full transition from the $SU(4)$ to $SU(2)$ transport regimes in a controllable way [20, 21]. In the present study, we are more interested in checking the robustness of the suppression of the $\mathcal{P}$ lines by inspecting the magnetic field evolution in different quadruplets. In general, neither in the quadruplets with stronger Kondo correlations (shells $N_\text{h}+1$ and $N_\text{h}+2$) nor in the one with a weaker Kondo effect (shell $N_\text{h}-1$), the $\mathcal{P}$ transition can be resolved from an analysis of the excitation spectrum, as exemplarily shown in the Supplementary Figure 8. Notice that due to gate voltage instability we do not report on the magnetic field evolution in the 1h valley of the N_h +1 and N_h+2 quadruplets.

Supplementary References

[1] Choi, M.-S., López, R. & Aguado, R. SU(4) Kondo effect in carbon nanotubes. *Phys. Rev. Lett.* **95**, 067204 (2005).

[2] Anders, F., Logan, D., Galpin, M. & Finkelstein, G. Zero-bias conductance in carbon nanotube quantum dots. *Phys. Rev. Lett.* **100**, 086809 (2008).

[3] Lim, J. S., Choi, M.-S., Choi, M. Y., López, R. & Aguado, R. Kondo effects in carbon nanotubes: From SU(4) to SU(2) symmetry. *Phys. Rev. B* **74**, 205119 (2006).

[4] Galpin, M. R., Jayatilaka, F. W., Logan, D. E. & Anders, F. B. Interplay between Kondo physics and spin-orbit coupling in carbon nanotube quantum dots. *Phys. Rev. B* **81**, 075437 (2010).

[5] Mantelli, D., Moca, C., Zaránd, G. & Grifoni, M. Kondo effect in a carbon nanotube with spin-orbit interaction and valley mixing: A DM-NRG study. *Physica E* **77**, 180-190 (2016).

[6] Oliveira, Wanda C. & Oliveira, Luiz N. Generalized numerical renormalization-group method to calculate the thermodynamical properties of impurities in metals. *Phys. Rev. B* **49**, 11986 (1994).

[7] Merker, L., Weichselbaum, A.,& Costi, T. Full density-matrix numerical renormalization group calculations of impurity susceptibility and specific heat of the Anderson impurity model. *Phys. Rev. B* **86**, 075150 (2012).

[8] Merker, L., & Costi, T. Numerical renormalization group calculations of impurity internal energy and specific heat of quantum impurity models. *Phys. Rev. B* **86**, 075153 (2012).

[9] Filippone, M.,Moca, C.P., Zaránd, G., & Mora, C. Kondo temperature of SU(4) symmetric quantum dots. *Phys. Rev. B* **90**, 121406 (2014).

[10] Grabert, H. & Devoret editors, M. *Single charge tunneling* (Plenum Press, New York, 1992).

[11] Koller, S., Leinsje, M., Wegewijs, M. & Grifoni, M. Density operator approaches to transport through interacting quantum dots: Simplifications in fourth-order perturbation theory. *Phys. Rev. B* **82**, 235307 (2010).

[12] König, J., Schmid, J., Schoeller, H. & Schön, G. Transport through an Anderson quantum dot in the intermediate coupling regime. *Phys. Rev. B* **54**, 16820-16837 (1996).

[13] Pedersen, J. N. & Wacker, A. Tunneling through nanosystems: Combining broadening with many-particle states. *Phys. Rev. B* **72**, 195330 (2005).

[14] Kern, J. & Grifoni, M. Transport through an Anderson quantum dot in the intermediate coupling regime. *Eur. Phys. J. B* **86**, 384 (2013).

[15] Dirnaichner, A. *et al.* Transport across a carbon nanotube quantum dot contacted with ferromagnetic leads: Experiment and nonperturbative modeling. *Phys. Rev. B* **91**, 195402 (2015).

[16] Toth, A., Moca, C., Legeza, Ö. & Zaránd, G. Density matrix numerical renormalization group for non-Abelian symmetries. *Phys. Rev. B* **78**, 245109 (2008).

[17] Smirnov, S. & Grifoni, M. Keldysh effective action theory for universal physics in spin-1/2 Kondo dots. *Phys. Rev. B* **87**, 121302(R) (2013).

[18] Schmid, D. *et al.* Broken SU(4) symmetry in a Kondo-correlated quantum dot. *Phys. Rev. B* **91**, 155435 (2015).

[19] Kretinin, A. V., Shtrikman, H. & Mahalu, D. Universal line shape of the Kondo zero-bias anomaly in a quantum dot. *Phys. Rev. B* **85**, 201301(R) (2012).

[20] Makarovski, A., Zhukov, A., Liu, J. & Finkelstein, G. SU(2) and SU(4) Kondo effects in carbon nanotube quantum dots. *Phys. Rev. B* **75**, 241407 (2007).

[21] Cleuziou, J. P., N'Guyen, N. V., Florens, S. & Wernsdorfer, W. Interplay of the Kondo effect and strong spin-orbit coupling in multihole ultraclean carbon nanotubes. *Phys. Rev. Lett.* **111**, 136803 (2013).
